# Link Prediction in Complex Networks Using Recursive Feature Elimination and Stacking Ensemble Learning

**DOI:** 10.3390/e24081124

**Published:** 2022-08-15

**Authors:** Tao Wang, Mengyu Jiao, Xiaoxia Wang

**Affiliations:** 1 School of Mathematics and Physics, North China Electric Power University, Baoding 071003, China; 2 Hebei Key Laboratory of Physics and Energy Technology, North China Electric Power University, Baoding 071000, China; 3 School of Control and Computer Engineering, North China Electric Power University, Baoding 071003, China

**Keywords:** ensemble learning, stacking, recursive feature elimination, link prediction, complex networks

## Abstract

Link prediction is an important task in the field of network analysis and modeling, and predicts missing links in current networks and new links in future networks. In order to improve the performance of link prediction, we integrate global, local, and quasi-local topological information of networks. Here, a novel stacking ensemble framework is proposed for link prediction in this paper. Our approach employs random forest-based recursive feature elimination to select relevant structural features associated with networks and constructs a two-level stacking ensemble model involving various machine learning methods for link prediction. The lower level is composed of three base classifiers, i.e., logistic regression, gradient boosting decision tree, and XGBoost, and their outputs are then integrated with an XGBoost model in the upper level. Extensive experiments were conducted on six networks. Comparison results show that the proposed method can obtain better prediction results and applicability robustness.

## 1. Introduction

Complex networks can be used to model various real-world complex systems such as social, biological and information systems, where nodes denote different individuals or entities in the system, and links or edges indicate the relations or interaction between nodes [1]. Great efforts have been devoted to analyze the network topologies, and predict links among nodes in order to better understand the evolution of networks. A challenging issue in complex network analysis is link prediction, which aims to reveal potential links or forecast future relations based on the known information of nodes and network structure [2,3].

A large number of methods have been proposed to solve the link prediction problem [4,5,6,7], which are mainly divided into similarity-based methods and learning-based methods. Similarity-based methods are the most common, and especially structural similarity, due to simple and efficient computation, and universal applicability to networks with similar structures. These methos hold the view that the probability of link existence between two nodes is proportional to their topological similarity. In terms of the topological information range used in similarity calculation, these methods can be grouped into three categories: local similarity methods, global similarity methods, and quasi-local similarity methods. Local similarity methods explore local information of the disconnected nodes such as common neighbors and node degree, and include Common Neighbors (CNs) [8], Jaccard coefficient (JC) [9], Preferential Attachment (PA) [10], the Adamic–Adar (AA) index [11], Resource Allocation (RA) [12], Cosine similarity, and the Salton index (SI) [13]. These methods achieve good results in many cases due to lower computational complexity and simple implementation [14]. Global similarity methods utilize the topological information of the whole network for link prediction. Klein et al. [15] introduced average commuting time (ACT) as a novel distance function, and considered that the node similarity is related to average commuting time. Brin et al. [16] presented the random walk with restart (RWR) index, an extended application of the PageRank algorithm, to improve the search efficiency of large-scale Web search engines. Jeh et al. [17] estimated the similarity score by considering recursively the similarity of neighboring nodes, and defined the SimRank index. These methods incur a higher computational cost to learn the global information from the known networks, and thus are infeasible for large networks. Quasi-local similarity methods have been introduced as trade-offs between local and global approaches, with the aim of extracting the same amount of topological information of networks as global methods and having the computing efficiency of local methods. Examples of such methods include local path (LP) [18], local random walk (LRW) [19], and superposed random walk (SRW) [19], whose results are a balance of prediction accuracy and computational complexity.

Learning-based methods, by comparison, predict the link likelihood of node pairs on the basis of machine learning algorithms. Specially, link prediction is treated as a binary classification problem, in which a prediction model is built by learning the topological features extracted from the observed network structure. Wu et al. [20] employed the AdaBoost algorithm to integrate CN, JC, AA, and RWR to predict links on two networks. Li et al. [21] aggregated logistic regression and XGBoost to learn four similarity indices to predict missing links. Ma et al. [22] used logistic regression to determine the weights of different similarity indices, and then predicted the connection probabilities of missing links. The ordered weighted averaging operator [23] and Choquet fuzzy integral [24] were utilized to fuse similarity indices. Furthermore, network embedding [25] and deep neural networks [26] were used to improve the performance of link prediction in complex networks. Learning-based methods usually perform better than similarity-based methods, but consume more resources in the training process of the prediction model [27].

Generally, different networks have their own structural features and one similarity index can only exploit some structural features of networks. One or several fixed similarity indices are applied to predict links on all networks, which may degrade the prediction performance. To this end, we incorporate the inherent topological features into link prediction for different networks. In this paper, a novel link prediction method is proposed, which combines random forest-based recursive feature elimination and stacking ensemble learning for link prediction, named RF-RFE-SELLP. The main contributions are three-fold. First, recursive feature elimination in random forest is employed to effectively select representative and relevant structural features associated with the networks. Second, a stacking ensemble framework is presented to combine the advantages of different machine learning methods to enhance the prediction results of the model. Considering better generalization performance and discrepancy of models, three models, i.e., logistic regression (LR), gradient boosting decision tree (GBDT), and XGBoost, are selected as the base models and XGBoost is employed as the top-level model. Third, extensive experiments on a variety of networks were carried out, showing that the proposed RF-RFE-SELLP method obtains better prediction results and applicability robustness than other comparison methods.

The remainder of this paper is organized as follows. Section 2 introduces the problem description, similarity indices, and datasets. The proposed method is presented in Section 3. Extensive experimental results are shown and analyzed in Section 4. Finally, Section 5 concludes this paper.

## 2. Preliminaries

### 2.1. Problem Description

Consider an undirected and unweighted network $G\left(V,E\right)$, where V and E denote the sets of nodes and links, respectively. As is usual, self-loops and multiple links are not allowed. For a network with *N* nodes, $A={\left\{a_{ij}\right\}}_{N\times N}$ represents the adjacency matrix. Let S be the universal set of all $\frac{N\left(N-1\right)}{2}$ possible links, and S−E be the set of nonexistent links. For each nonexistent link $e\left(v_{i},v_{j}\right)\in S-E,v_{i},v_{j}\in V$, the similarity score is evaluated to quantify the link existence likelihood according to a defined similarity index. Generally, the link set *E* can be randomly divided into two parts: training set $E^{T}$ and test set $E^{P}$, where $E=E^{T}\cup E^{P}$, $E^{T}\cap E^{P}=\emptyset$. The training set $E^{T}$ is utilized to compute the similarity index to obtain the similarity score of the test set $E^{P}$. Link prediction methods exploit the known topological information to predict the missing or unknown links in the network. It can be deemed to be a binary classification problem, where the class label is determined by the existence of links. If there is a direct connection between two nodes, the label is 1; otherwise, the label is 0. In this way, each link is assigned a label, and then the topological features of each link are extracted from the network information by computing the similarity indices. The goal of link prediction is to utilize these extracted topological features to forecast the links in the network *G*.

### 2.2. Similarity Indices

Let $\Gamma_{v_{i}}$ denote the neighbor set of node $v_{i}$, and $k_{v_{i}}$ denote its degree. Several similarity indices are introduced as follows.

(i) Common Neighbor (CN) index [8]: the CN index emphasizes that a pair of nodes with more common neighbors are more likely to be connected:

(1) $$S^{CN}\left(v_{i},v_{j}\right)=\left|\Gamma_{v_{i}}\cap \Gamma_{v_{j}}\right|$$

(ii) Preferential Attachment (PA) index [10]: the PA index assumes that the connection probability of a pair of nodes is proportional to the product of their degree:

(2) $$S^{PA}\left(v_{i},v_{j}\right)=k_{v_{i}}\cdot k_{v_{j}}$$

(iii) Adamic–Adar (AA) index [11]: the AA index assigns more weights to the common neighbor nodes with a low degree:

(3) $$S^{AA}\left(v_{i},v_{j}\right)=\sum_{v_{l}\in \Gamma_{v_{i}}\cap \Gamma_{v_{j}}}\frac{1}{lg\left(k_{v_{l}}\right)}.$$

(iv) Leicht–Holme–Newman (LHN) index [28]: a pair of nodes with more common neighbors has high similarity, and the product of two node degrees is proportional to the mean of a common neighbor’s number:

(4) $$S^{LHN}\left(v_{i},v_{j}\right)=\frac{\left|\Gamma_{v_{i}}\cap \Gamma_{v_{j}}\right|}{k_{v_{i}}\times k_{v_{j}}}$$

(v) Resource Allocation (RA) index [12]: the proposal of RA index was inspired by the process of network resource allocation. Some resources are passed by node $v_{i}$ to $v_{j}$, and their common neighbor is the transmission medium. The number of resources received by $v_{j}$ is defined as the similarity:

(5) $$S^{RA}\left(v_{i},v_{j}\right)=\sum_{v_{l}\in \Gamma_{v_{i}}\cap \Gamma_{v_{j}}}\frac{1}{k_{v_{l}}}.$$

(vi) Average Commute Time (ACT) index [15]: the ACT index assumes that the smaller the average commute time of two nodes, the closer the two nodes. Denote $m\left(x,y\right)$ as the average number of steps that a random walker starting from node $v_{i}$ to reach node $v_{j}$. The average commute time is then described as:

(6) $$n\left(i,j\right)=m\left(i,j\right)+m\left(j,i\right)$$

By solving the pseudoinverse of the Laplacian matrix *L*^+^, we can obtain:

(7) $$n\left(i,j\right)=M\left(l_{ii}^{+}+l_{jj}^{+}-2l_{ij}^{+}\right)$$

and then the similarity is defined as:

(8) $$S^{ACT}\left(v_{i},v_{j}\right)=\frac{1}{l_{ii}^{+}+l_{jj}^{+}-2l_{ij}^{+}}$$

where $l_{ij}^{+}$ represents the corresponding element in the matrix $L^{+}$.

(vii) Matrix Forest Index (MFI) index [29]: the MFI index is based on matrix-forest theory and expressed as:

(9) $$S^{MFI}\left(v_{i},v_{j}\right)={\left(1+\alpha L\right)}^{-1},\alpha >0$$

where *L* is the Laplacian matrix.

(viii) Random Walk with Restart (RWR) index [16]: the RWR index assumes that the random walker returns to the starting point with a certain probability 1 − *c*, and P is the transition probability matrix. The probability vector of arriving at each node $v_{i}$ of the network at time *t* + 1 is:

(10) $$\pi_{v_{i}}\left(t+1\right)=c\cdot P^{T}\pi_{v_{i}}\left(t\right)+\left(1-c\right)e_{v_{i}}$$

where $e_{v_{i}}$ represents the starting point, and the similarity index is defined as:

(11) $$S^{RWR}\left(v_{i},v_{j}\right)=\pi_{v_{i}v_{j}}+\pi_{v_{j}v_{i}}$$

where $\pi_{v_{i}v_{j}}$ represents the probability from node $v_{i}$ to node $v_{j}$.

(ix) SimRank index [17]: the SimRank index states that two nodes are similar if they are connected to similar nodes:

(12) $$S^{SimRank}\left(v_{i},v_{j}\right)=C\cdot \frac{\sum_{v_{l}\in \Gamma_{v_{i}}}\sum_{v_{l}^{\prime }\in \Gamma_{v_{j}}}s_{v_{l}v_{l}^{\prime }}^{SimRank}}{k_{v_{i}}\cdot k_{v_{j}}}$$

where the attenuation parameter $s_{v_{i}v_{i}}=1$, and $C\in \left[0,1\right]$.

(x) Local Path (LP) index [18]: the LP index considers local paths on the basis of the CN index, and attains a balance between the precision and computational complexity:

(13) $$S^{LP}\left(v_{i},v_{j}\right)=A^{2}\left(v_{i},v_{j}\right)+\alpha A^{3}\left(v_{i},v_{j}\right)$$

where $A^{2}\left(v_{i},v_{j}\right)$ and $A^{3}\left(v_{i},v_{j}\right)$ are the number of different paths with the length 2 and 3, respectively, and α is the free parameter α=0.001.

(xi) Local Random Walk (LRW) index [19]: the random walker starts from node $v_{i}$ at time *t*, and $\pi_{v_{i}v_{j}}\left(t\right)$ is the probability that the walker just goes to node $v_{j}$ at time *t* + 1:

(14) $$\pi_{v_{i}}\left(t+1\right)=P^{T}\pi_{v_{i}}\left(t\right),t\geq 0$$

The initial resource of node $v_{i}$ is $q_{v_{i}}$, and the LRW index at time step *t* is defined as:

(15) $$S^{LRW}\left(t\right)=q_{v_{i}}\pi_{v_{i}v_{j}}\left(t\right)+q_{v_{j}}\pi_{v_{j}v_{i}}\left(t\right)$$

(xii) Superposed Random Walk (SRW) index [19]: on the basis of the LRW index, the value of the SRW index can be acquired by summing the results of step *t* and the previous step:

(16) $$S^{SRW}\left(t\right)=\sum_{\tau =1}^{t}S^{LRW}\left(\tau \right)=\sum_{\tau =1}^{t}\left[q_{v_{i}}\pi_{v_{i}v_{j}}\left(\tau \right)+q_{v_{j}}\pi_{v_{j}v_{i}}\left(\tau \right)\right]$$

### 2.3. Dataset Description

To evaluate the effectiveness of the proposed RF-RFE-SELLP model, we performed massive experiments on six real-world networks from different fields. The basic statistics of these networks are summarized in Table 1 and a brief description is provided.

*C. elegans* [30]: The Caenorhabditis elegans network consists of 297 neurons and 2148 connections.

Vicker [31]: the Vicker dataset is a social network between seventh grade students in a school in Victoria, Australia., which consists of 29 students and 376 links.

Email [32]: the E-mail network has 1133 users and 5451 communications between the members of University at Rovira i Virgili.

NS [33]: the NetScience dataset is a co-authorship network consisting of 1589 scientists and 2742 co-authorships.

SciMet [34]: the SciMet dataset is a network of articles from or cited by Scientometrics that includes 3084 articles and 10,399 links.

Router [35]: the Router dataset is an internet router hierarchical network containing 5022 routers and 6258 links.

## 3. Proposed Framework: RF-RFE-SELLP

In this section, the proposed RF-RFE-SELLP model is presented in detail, including model structure and algorithm procedure.

### 3.1. Motivation

Twelve similarity indices are introduced in Section 2.2, among which each index exploits one or two structural features of networks [36]. The fusion of these indices can incorporate multiple structural features to improve prediction performance. However, different networks have different inner structural features.

To validate this fact, a series of experiments was constructed on the six networks described above. For each network, the matching score [22] of one similarity index is computed:

(17) $$\sigma =\frac{\left|E\cap \tilde{E}\right|}{\left|E\right|}$$

where $\tilde{E}$ denotes the set of top $\left|E\right|$ ranked links predicted by the predefined index. The larger the value of σ, the better the accuracy of the index. In this work, the PA index and the LP index are employed to illustrate that one similarity index plays different roles in different networks. The matching scores of the two indices, $\sigma_{PA}$ and $\sigma_{LP}$, respectively, are calculated according to Equation (17), and the difference between them is defined as:

(18) $$\Delta \sigma =\sigma_{LP}-\sigma_{PA}$$

**Figure 1:**
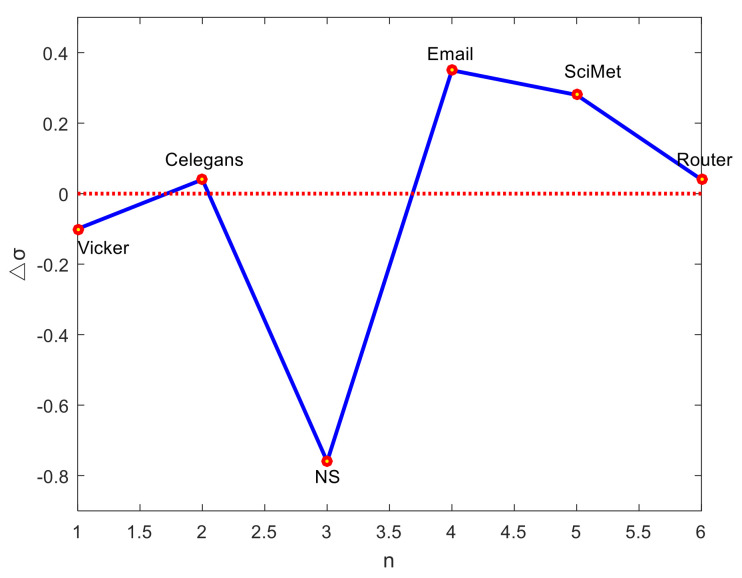
Difference in matching scores between the LP index and the PA index.

The values of Δσ for the six networks are shown in Figure 1. It can be observed that for NS and Vicker networks, the role of the PA index is superior to that of the LP index; that is, the structural features induced by the PA index can better represent this network. For the Email and SciMet networks, the role of the LP index is greater than that of the PA index, which indicates that LP feature is more representative on these networks. For the remaining two networks, there is little difference between the two indices. Therefore, it is unwise to use structural features of one similarity index for link prediction in all networks. Due to different contributions of each index to different networks, it is essential to select suitable features of networks to improve the prediction accuracy.

### 3.2. Random Forest-Based Recursive Feature Elimination for Feature Selection

In random forest (RF), feature selection can be performed in a very simple way by the removal of features with low importance. However, RF can only provide the ranking of importance for each feature, but cannot determine the effective features and the number of features in the optimal feature set. In order to choose the relevant features for building the classification model, we recursively remove the features with lower importance to obtain smaller feature subsets, estimate the discriminative abilities of features of the subsets, and select those features with greatest discriminative power to enhance the prediction performance.

For *m* features of $x_{1},\;x_{2},\;\cdots ,\;x_{m}$, the variable importance measure (VIM) is computed to evaluate the importance of each feature, and can be derived from the Gini index. The Gini index can be defined as follows:

(19) $$GI_{t}=\sum_{c=1}^{C}\sum_{c^{\prime }\neq c}p_{tc}p_{tc^{\prime }}=1-\sum_{c=1}^{C}p_{tc}^{2}$$

where *C* represents the number of categories, and $p_{tc}$ indicates the proportion of samples that belongs to category *c* for node *t*. The VIM of feature $x_{j}$ in node *t* is:

(20) $$VIM_{jt}^{node}=GI_{m}-GI_{l}-GI_{r}$$

where $GI_{l}$ and $GI_{r}$ represent the Gini index of left and right child branches, respectively. The VIM of feature $x_{j}$ in the *i*-th decision tree is:

(21) $$VIM_{ji}^{tree}=\sum_{t\in T}VIM_{jt}$$

where *T* is the node set of feature $x_{j}$ in the *i*-th decision tree. The VIM of feature $x_{j}$ in RF is:

(22) $$VIM_{j}^{RF}=\sum_{i=1}^{n}VIM_{ij}$$

where *n* is the number of trees in RF. The importance score of feature $x_{j}$ is defined by normalizing the $VIM_{j}$:

(23) $$VIM_{j}=\frac{VIM_{j}}{\sum_{i=1}^{12}VIM_{i}}$$

The higher the value of $VIM_{j}$, the greater the importance of feature $x_{j}$ on RF for classification.

Furthermore, recursive feature elimination (RFE) [37] is utilized to repeatedly remove the feature with the lowest VIM to obtain a new feature subset until only one feature remains in the feature set. We estimate the discriminative abilities of features in the subsets and then select the optimal features. To find the optimal number of features, k-fold cross-validation is employed to score different feature subsets and select the best scoring set of features. The cross-validation score (CVS) is used to evaluate the discrimination of each feature subset, defined as:

(24) $$\mathrm{CVS}=\frac{\sum_{k=1}^{K}P_{k}}{K}$$

where $p_{k}$ is the classification accuracy on the *k*-th cross validation subset. The feature subset with the highest CVS is chosen as the optimal one. The proposed RF-RFE method aims to eliminate irrelevant features and redundant features to represent the original features with as few features as possible in order to improve the generalization ability of the classification model. In this work, *k* is set as 5, and the simulation is conducted 20 times.

### 3.3. Stacking Ensemble Learning for Link Prediction

**Figure 2:**
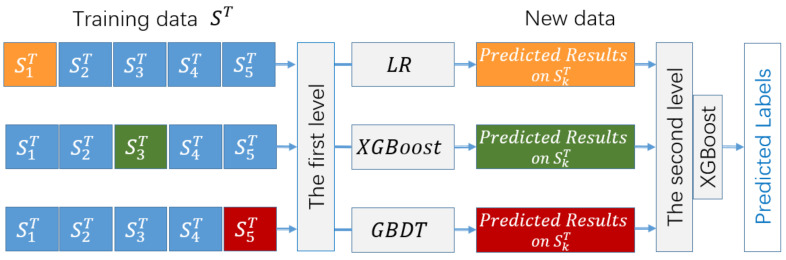
SELLP model.

Ensemble learning combines multiple weak supervised models to form a better supervised model. Ensemble learning methods are mainly divided into three categories: bagging, boosting, and stacking. Stacking fuses information from base models to generate a new model to obtain better classification performance than that of a single model. The base model with strong learning ability is conductive to improving the classification results. Moreover, the classification models with great differences should be selected as the base model, which can reflect the advantages of different algorithms to the greatest extent. In this paper, a stacking ensemble learning model for link prediction is proposed, named SELLP, in which three different models, i.e., XGBoost, LR, and GBDT, are selected as the base models, and XGBoost, which has better generalization performance, is employed as the top-level model. The model is shown in Figure 2.

For a network with *N* nodes, a dataset S is constructed containing all possible links. The dataset S is divided into a training set $S^{T}$ and test set $S^{P}$, where $S^{T}=\left\{\left(x_{i},y_{i}\right),i=1,\ldots ,N^{T}\right\}$, $N^{T}$ is the number of samples, $x_{i}\in {\mathbb{R}}^{D}$, and $y_{i}$ is the feature vector and label of the *i*-th sample, respectively. In the training phase, the dataset $S^{T}$ is randomly divided into five subsets $S_{k}^{T},\;k=1,\ldots ,5$ with the same size. Let $S_{-k}^{T}=S^{T}-S_{k}^{T}$, $S_{-k}^{T}$, and $S_{k}^{T}$ be the *k*-th training set and test set in the 5-fold cross validation, respectively. For the base model $C_{j},j=1,2,3$ of the first layer, the training set $S_{-k}^{T}$ is used to train each base model $C_{j}$ to obtain the base model $C_{jk},\;k=1,\ldots ,5$. After cross validation, 3×5=15 base models are obtained. For each sample $x_{i}$ in the *k*-th test set $S_{k}^{T}$, the prediction result of the base model $C_{jk}$ is ${\hat{y}}_{ji}$. The probability predicted by each base model $C_{j}$ on the subset $S_{k}^{T}$ forms a new dataset $S_{new}^{T}=\left\{\left({\hat{y}}_{1i},{\hat{y}}_{2i},{\hat{y}}_{3i},y_{i}\right),i=1,\ldots ,N^{T}\right\}$. The newly generated dataset is then used to train the fuse model of the second layer in the SELLP model. By learning from the new dataset $S_{new}^{T}$, the XGBoost model can fuse the learning results of multiple base models in the first layer.

For each base model, the test data are different from the training data. All the data are utilized only once in the training phase, which can effectively prevent overfitting. Moreover, in the SELLP model, the training results of base models in the first layer are fully used in the induction process of the fusion model in the second layer. The fusion model can optimize and correct the prediction results of base models in the first layer to improve the accuracy of the SELLP model.

In the test phase, each sample in the test set $S^{P}=\left\{x_{l},l=1,\ldots ,N^{P}\right\}$ is fed into the base models in the first layer. For the sample $x_{l}$, the base models $C_{jk}$, k=1,…,5, predict the classification results ${\tilde{y}}_{jkl}$, respectively, and then average classification results ${\tilde{y}}_{jl}=\frac{1}{5}\sum_{k=1}^{5}{\tilde{y}}_{jkl}$ can be obtained for each base model $C_{j}$. The classification results of three base models form a data $\left({\tilde{y}}_{1l},{\tilde{y}}_{2l},{\tilde{y}}_{3l}\right)$, which is sent to the fuse model to obtain the final classification results given by the SELLP model.

### 3.4. RF-RFE-SELLP Algorithm

The training procedure of the proposed RF-RFE-SELLP model is summarized as follows:

Step 1: Obtain the adjacency matrix of a network and calculate the similarity scores of 12 feature indices to construct the feature vectors of links in the network.

Step 2: Estimate the VIM of features by the Gini index in random forest, then remove the feature with the lowest VIM, and calculate the corresponding CVS to obtain the optimal feature subset.

Step 3: Construct the dataset including the features and labels of all links, and divide this dataset into the training set and test set. LR, XGBoost, and GBDT are used as the base models in the first layer. Train the base models separately on the training set by 5-fold cross validation, and then obtain the classification results of each base model.

Step 4: Construct new training data from the prediction results by the base models and utilize these data to train the fuse model in the second layer. Then, the final RF-RFE-SELLP model can be obtained. The specific steps of RF-RFE-SELLP algorithm are presented in Algorithm 1.
**Algorithm 1: RF-RFE-SELLP algorithm**Input: Network $G=\left(V,E\right)$ Output: Parameters of SELLP model 1: Calculate the adjacency matrix A of network *G*. 2: for each $v_{i},v_{j}\in V$ do 3: $y_{n}\leftarrow A\left(:\right)$
4: $x_{n}\leftarrow \left(x^{S_{1}}\left(v_{i},v_{j}\right),\ldots ,x^{S_{12}}\left(v_{i},v_{j}\right)\right)$
5:/*$y_{n}$ is the label, $x_{n}$ is the feature vector, and $x^{S_{l}}\left(v_{i},v_{j}\right)$ is the score computed by the similarity index $S_{l}$.*/ 6: end for 7: for r←1 to 12 do 8: Compute the VIM of features, and remove the feature with the lowest one. 9: Update feature ranked list, and calculate the corresponding CVS. 10: end for 11: Obtain the optimal feature subset $F_{n}$ by comparing CVS. 12: Construct the set S including the feature subset $F_{n}$ and label $y_{n}$, and then divide S into the training set $S^{T}$ and test set $S^{P}$. 13: for k←1 to 5 do 14: for j←1 to 3 do 15: Train the base model $C_{jk}$ on the training set $S_{-k}^{T}$. 16: Predict the probability ${\hat{y}}_{jk}$ on the test set $S_{k}^{T}$. 17: end for 18: end for 19: Construct the set $S_{new}^{T}=\left\{\left({\hat{y}}_{1i},{\hat{y}}_{2i},{\hat{y}}_{3i},y_{i}\right),i=1,\ldots ,N^{T}\right\}$ by the prediction results of each base model. 20: Train the fuse model of the second layer on the set $S_{new}^{T}$. 21: Return the parameters of RF-RFE-SELLP model.

## 4. Experiments

In this section, we compare the proposed RF-RFE-SELLP with similarity-based methods described in Section 2 and supervised learning-based methods, i.e., multi-layer perceptron neural networks (MLP), RF, and AdaBoost for the purpose of validating the performance of RF-RFE-SELLP.

### 4.1. Experimental Setting

Due to the sparsity of real networks, the training set and test set should be generated with balanced class distribution. The number of existent links is calculated, and the same number of data is randomly selected from the nonexistent links to form a new dataset D. We randomly select a certain fraction (90%) of links from D as the training set $D^{T}$, and the remaining data as the test set $D^{P}$.

Three supervised-learning methods, namely AdaBoost, RF and MLP, were employed to identify missing links in the following experiments. The grid search and cross validation are utilized to determine the parameters of the models. All of the link prediction methods were coded in Python 3.8 and all experiments were implemented on a computer with an Intel(R) Xeon(R) Silver 4214 CPU and 32 GB memory. All experimental results are the average results of 10 independent runs.

### 4.2. Evaluation Metric

In this paper, link prediction is regarded as a binary classification problem. The label of a node pair is positive if a link exists between them, and negative otherwise. Four common terms are introduced to formulate the evaluation metrics [38]. True Positive (TP) is the number of node pairs with links that are correctly recognized as positive. False Positive (FP) corresponds to the number of node pairs without links that are incorrectly labeled as positive. False Negative (FN) refers to the number of node pairs with links that are incorrectly as identified negative. True Negative (TN) is the number of node pairs without links that are correctly classified as negative.

Four evaluation metrics are employed in this work to quantify the prediction performance of algorithms, i.e., Accuracy, Precision, F1-score, and AUC. Accuracy is the proportion of all correctly classified node pairs in the set of node pairs. Precision represents the proportion of all node pairs with links in the set of node pairs classified as positive. Recall is the proportion of node pairs correctly classified as positive in the set of node pairs with links. F1-score is the harmonic average of Precision and Recall.

(25) $$\mathrm{Accuracy}=\frac{\mathrm{TP}+\mathrm{TN}}{\mathrm{TP}+\mathrm{FP}+\mathrm{TN}+\mathrm{FN}}$$

(26) $$\mathrm{Precision}=\frac{\mathrm{TP}}{\mathrm{TP}+\mathrm{FP}}$$

(27) $$\mathrm{Recall}=\frac{\mathrm{TP}}{\mathrm{TP}+\mathrm{FN}}$$

(28) $$F1-\mathrm{score}=\frac{2\mathrm{Precision}\cdot \mathrm{Recall}}{\mathrm{Precision}+\mathrm{Recall}}$$

Area under the curve (AUC) can describe the overall performance of the prediction model, given as:

(29) $$\mathrm{AUC}=\frac{S_{0}-n_{0}\left(n_{0}+1\right)/2}{n_{0}n_{1}}$$

where $n_{0}$ and $n_{1}$ are the numbers of positive and negative node pairs, respectively, and $S_{0}=\sum r_{i}$, $r_{i}$ is the rank of *i*-th positive node pairs in the ranked list.

### 4.3. Performance Comparison

**Figure 3:**
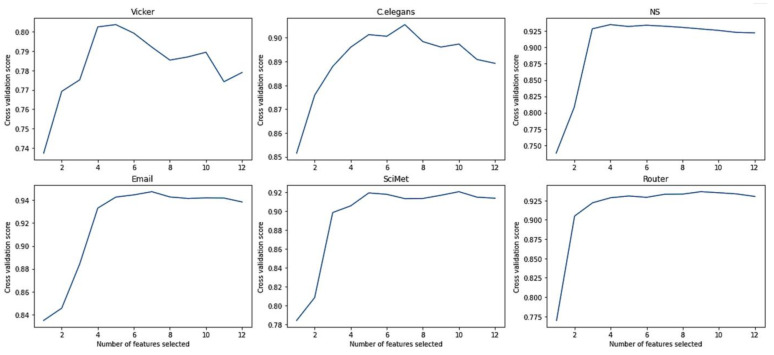
The CVS of selected features.

Figure 3 shows the CVS of selected features obtained by RF-RFE on six networks, where the abscissa indicates the number of selected features and the vertical ordinate represents the corresponding cross validation score. It can be observed that the CVS increases and gradually stabilizes as the number of selected features increases for most networks. The higher CVS indicates the higher discriminative ability of features. In this way, the optimal feature subset can be determined with fewer features and a higher CVS. Taking the Vicker network, for instance, the value of CVS increases from 73.73% to a maximum of 80.36% when the number of features increases from one to five. When the number of features is greater than five, there is a slight decrease in the CVS. This indicates that too many features do not necessarily produce higher classification accuracy. The five features are selected for the Vicker network that make significant contributions to classification results.

To investigate module-wise effectiveness of the three base models and feature selection on the overall performance, the ensemble model, i.e., SELLP (RF-RFE-SELLP without RF-RFE feature selection), and three base models, i.e., GBDT, XGBoost, and LR, are evaluated, respectively. Table 2, Table 3, Table 4 and Table 5 summarize the performance in terms of AUC, Accuracy, Precision, and F1-score of the six networks. With the same features, the ensemble model exhibits better results than the three base models in terms of the four metrics, which highlights the advantage of stacking ensemble learning. Each classification model with the selected features by RF-RFE can achieve better performance results than that with the original features, which proves the effectiveness of RF-RFE feature selection. RF-RFE can eliminate irrelevant and redundant features to represent the raw data in order to improve the generalization ability of the classification model. Moreover, the proposed RF-RFE- SELLP outperforms the comparison methods on all of the six networks. In detail, our model exhibits the highest AUC of 0.9118, Accuracy of 0.8985, Precision of 0.8743, and F1-score of 0.9156 on the Vicker network. Similar results can be observed on the other networks.

**Figure 4:**
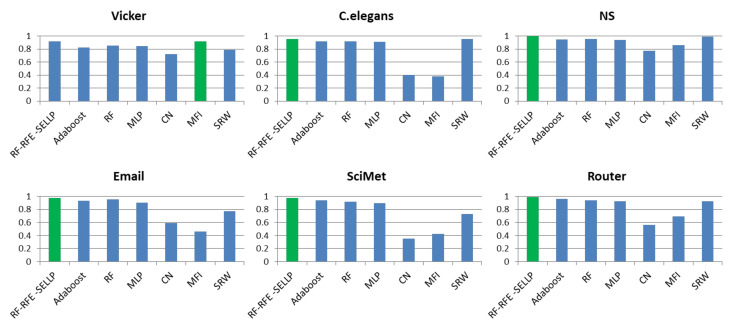
AUC results of all comparative methods on six networks. The best performance is emphasized in green for each network.

**Figure 5:**
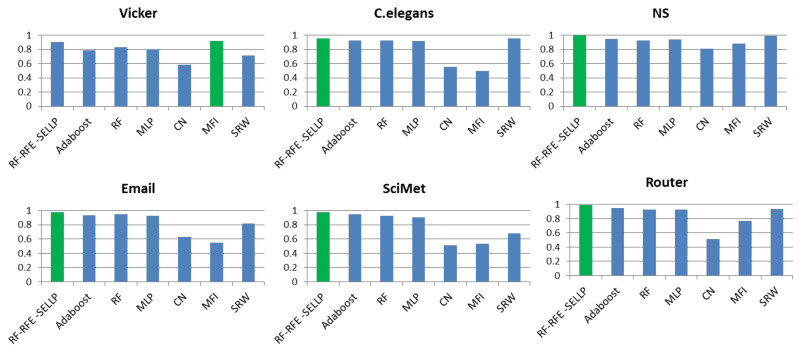
Accuracy results of all comparative methods on six networks. The best performance is emphasized in green for each network.

**Figure 6:**
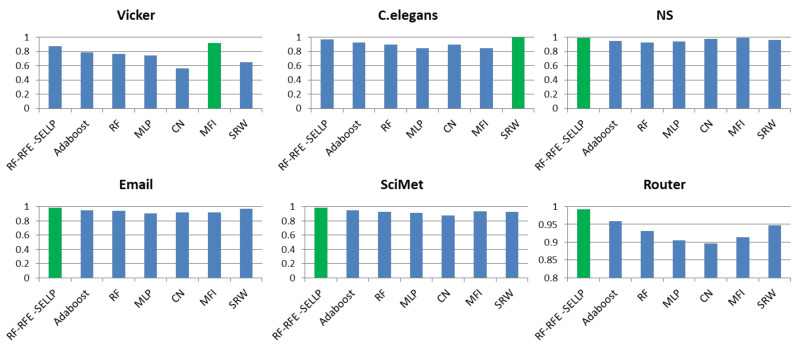
Precision results of all comparative methods on six networks. The best performance is emphasized in green for each network.

**Figure 7:**
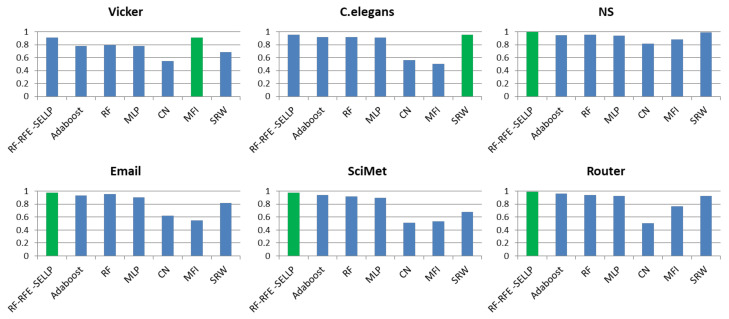
F1-score results of all comparative methods on six networks. The best performance is emphasized in green for each network.

To further evaluate the performance of the proposed method, the RF-RFE-SELLP method is compared with supervised learning-based methods, i.e., AdaBoost, RF, and MLP, and similarity-based methods, i.e., CN, MFI, and SRW. Three chosen similarity-based methods belong to global similarity, local similarity, and quasi-local similarity, respectively. Figure 4, Figure 5, Figure 6 and Figure 7 illustrate the comparison results regarding AUC, Accuracy, Precision, and F1-score on the six networks, where one bar with green color indicates the best result on this network.

It can be seen that RF-RFE-SELLP outperforms all of the comparison methods in terms of the four metrics on four networks, i.e., NS, Email, SciMet, and Router. For the Vicker network, the MFI index performs best in the four metrics, whereas the SRW index achieves the best results in terms of AUC and Precision for the *C. elegans* network. Although some similarity indices can obtain good performance on some networks, the performance varies greatly on different networks. The Precision of the CN index is 0.9710 on the NS network and 0.5606 on the Vicker network; the F1-score of the MFI index is 0.9189 on the Vicker network, but 0.3477 on *C. elegans* network; and the Precision of the SRW index is 0.9973 on the *C. elegans* network and 0.6491 on the Vicker network. Among the three similarity indices, the CN index achieves the worst performance on Vicker, NS, SciMet, and Router networks, and the worst results are obtained by the MFI index on *C. elegans* and Email networks. Compared with similarity-based methods, supervised learning-based methods can obtain more stable results in most respects on most networks. MLP yields the worst results in terms of Precision and Accuracy on *C. elegans*, NS, Email, SciMet, and Router networks, and the worst classification model is AdaBoost for the Vicker network.

Furthermore, we performed the statistical test for the proposed RF-RFE-SELLP and six comparative methods, i.e., AdaBoost, RF, MLP, CN, MFI, and SRW, in terms of AUC, Accuracy, Precision, and F1-score on six networks. Table 6 shows the results for Accuracy on the Email network.

The F-test and t-test were performed to compare the prediction results. In the case of RF, as a result of the F-test, the obtained *p*-value (0.45) was greater than the significance level of 0.05, indicating there is no difference in variance between RF-RFE-SELLP and RF. Then, a two-sample assuming equal variance t-test was conducted and the obtained *p*-value (3.41 × 10^−3^) was smaller than the significance level of 0.05; that is, there is a statistically significant difference in mean Accuracy between RF-RFE-SELLP and RF. In addition, the 95% confidence interval of mean difference does not include zero. From the results, we can conclude that RF-RFE-SELLP outperforms RF in Accuracy. A similar conclusion can be obtained for AdaBoost, MLP, CN, MFI, and SRW. Moreover, the experimental results in terms of AUC, Precision, and F1-score for these methods also lead to the same conclusion.

### 4.4. Robustness

**Figure 8:**
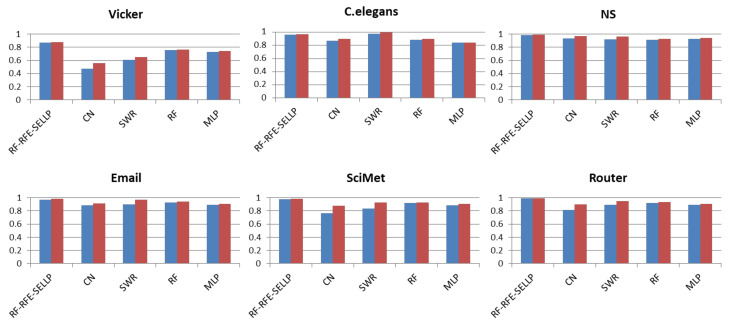
Precision results with different training set sizes.

**Figure 9:**
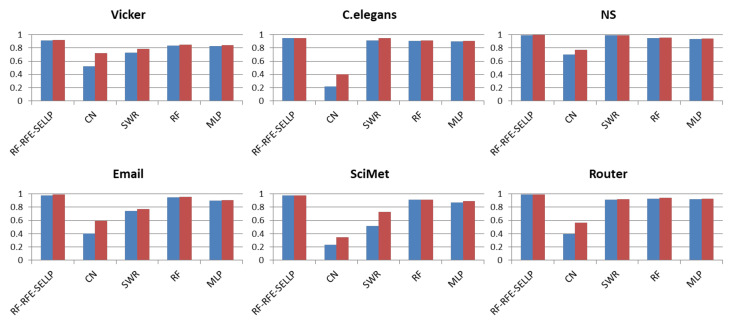
F1-score results with different training set sizes.

In order to verify the robustness of the proposed RF-RFE-SELLP to the size of the training set, we compared CN, SRW, RF, MLP, and RF-RFE-SELLP. We performed the experiments with 80% and 90% training sets. Figure 8 and Figure 9 show the variation in performance in terms of Precision and F1-score with training sets of different sizes, where one bar with blue color indicates the result with 80% training set and one with red color denotes the result with 90% training set. When we reduce the training set size from 90% to 80%, the performance degrades for all methods. However, the degradation is much smaller in the three supervised learning-based methods, i.e., RF, MLP, and RF-RFE-SELLP, compared to similarity-based methods, i.e., CN and SRW. Two indices work solely on local structural information of networks so that 10% removal of links affects their similarity scores, and the decline in F1-score is greater than that in Precision. Three supervised learning-based methods, especially RF-RFE-SELLP, select strong structural features from networks, which results in slight performance degradation.

## 5. Conclusions

The contributions of this paper include proposing the use of recursive feature elimination in random forest to choose representative structural features for networks, in addition to using the stacking ensemble learning method to predict the missing links. The RF-RFE-SELLP algorithm proposed in this paper is an ensemble learning framework that integrates LR, GBDT, and XGBoost models. Extensive experiments show that the performance of RF-RFE-SELLP in terms of AUC, Accuracy, Precision, and F1-score consistently exceeds that of the individual base model, and the comparison with the no feature selection method demonstrates encouraging performance in terms of these metrics. Moreover, the RF-RFE-SELLP method can achieve better performance than several supervised learning-based and similarity-based methods for most networks.

## Figures and Tables

**Table 1:** Basic statistics of six networks. *Notation: N* is the number of nodes, *M* is the number of links, *C* is the clustering coefficient, *R* is the assortative coefficient, and *H* is the degree heterogeneity.

Networks	*N*	*M*	*C*	*R*	*H*
*C. elegans*	297	2148	0.308	−0.163	1.800
Vicker	29	376	0.733	−0.157	0.982
Email	1133	5451	0.254	0.078	1.942
NS	1589	2742	0.791	0.462	2.011
SciMet	3084	10,399	0.175	−0.033	2.78
Router	5022	6258	0.033	−0.138	5.503

**Table 2:** AUC results of the base and ensemble models on six networks.

Results Based on the Original Features						
Methods	Vicker	*C. elegans*	NS	Email	SciMet	Router
SELLP	0.8290	0.9236	0.9538	0.9424	0.9378	0.9604
GBDT	0.7750	0.9079	0.9461	0.9308	0.9138	0.9477
XGBoost	0.7604	0.9085	0.9457	0.9460	0.9268	0.9531
LR	0.7318	0.8631	0.9365	0.9263	0.8967	0.9287
**Results based on the selected features using RF-RFE**						
**Methods**	**Vicker**	***C. elegans***	**NS**	**Email**	**SciMet**	**Router**
RF-RFE -SELLP	0.9118	0.9525	0.9949	0.9747	0.9764	0.9884
RF-RFE-GBDT	0.8663	0.9419	0.9747	0.9476	0.9438	0.9680
RF-RFE-XGBoost	0.8059	0.9278	0.9786	0.9521	0.9563	0.9769
RF-RFE-LR	0.8263	0.9121	0.9627	0.9497	0.9432	0.9581

**Table 3:** Accuracy results of the base and ensemble models on six networks.

Results Based on the Original Features						
Methods	Vicker	*C. elegans*	NS	Email	SciMet	Router
SELLP	0.8260	0.9246	0.9536	0.9524	0.9447	0.9600
GBDT	0.7681	0.9093	0.9245	0.9434	0.9237	0.9276
XGBoost	0.7826	0.9030	0.9453	0.9461	0.9279	0.9492
LR	0.7681	0.8813	0.9353	0.9361	0.9019	0.9248
**Results based on the selected features using RF-RFE**						
**Methods**	**Vicker**	***C. elegans***	**NS**	**Email**	**SciMet**	**Router**
RF-RFE-SELLP	0.8985	0.9534	0.9945	0.9747	0.9764	0.9884
RF-RFE-GBDT	0.8405	0.9369	0.9745	0.9611	0.9537	0.9780
RF-RFE-XGBoost	0.8405	0.9355	0.9854	0.9606	0.9572	0.9768
RF-RFE-LR	0.8115	0.9081	0.9726	0.9592	0.9328	0.9492

**Table 4:** Precision results of the base and ensemble models on six networks.

Results Based on the Original Features						
Methods	Vicker	*C. elegans*	NS	Email	SciMet	Router
SELLP	0.8246	0.9294	0.9545	0.9488	0.9481	0.9652
GBDT	0.7868	0.8919	0.9309	0.9375	0.9100	0.9474
XGBoost	0.7822	0.9097	0.9455	0.9419	0.9310	0.9523
LR	0.7791	0.8696	0.9370	0.9221	0.9098	0.9259
**Results based on the selected features using RF-RFE**						
**Methods**	**Vicker**	***C. elegans***	**NS**	**Email**	**SciMet**	**Router**
RF-RFE-SELLP	0.8743	0.9670	0.9927	0.9794	0.9853	0.9913
RF-RFE-GBDT	0.8614	0.9320	0.9727	0.9548	0.9510	0.9789
RF-RFE-XGBoost	0.8367	0.9469	0.9845	0.9592	0.9524	0.9744
RF-RFE-LR	0.8189	0.8934	0.9781	0.9481	0.9428	0.9576

**Table 5:** F1-score results of the base and ensemble models on six networks.

Results based on the original features						
Methods	Vicker	*C. elegans*	NS	Email	SciMet	Router
SELLP	0.8571	0.9213	0.9536	0.9421	0.9374	0.9601
GBDT	0.8048	0.9059	0.9457	0.9304	0.9136	0.9478
XGBoost	0.8314	0.9066	0.9455	0.9457	0.9265	0.9533
LR	0.8260	0.8552	0.9363	0.9257	0.8960	0.9290
**Results based on the selected features using RF-RFE**						
**Methods**	**Vicker**	***C. elegans***	**NS**	**Email**	**SciMet**	**Router**
RF-RFE-SELLP	0.9156	0.9501	0.9945	0.9744	0.9762	0.9885
RF-RFE-GBDT	0.8607	0.9380	0.9745	0.9473	0.9436	0.9682
RF-RFE-XGBoost	0.8817	0.9261	0.9785	0.9512	0.9559	0.9769
RF-RFE-LR	0.8395	0.9109	0.9624	0.9486	0.9429	0.9584

**Table 6:** Statistical test for RF-RFE-SELLP vs. AdaBoost, RF, MLP, CN, MFI, and SRW in mean Accuracy on the Email network.

	AdaBoost	RF	MLP	CN	MFI	SRW
*p*-value (F-test)	0.48	0.45	0.11	0.02	0.14	0.19
*p*-value (t-test)	4.95 × 10^−5^	3.41 × 10^−3^	1.25 × 10^−4^	3.14 × 10^−7^	4.56 × 10^−9^	6.09 × 10^−9^
Mean Accuracy	0.9341	0.9461	0.9249	0.6227	0.5441	0.8161
Mean Accuracy of RF-RFE-SELLP	0.9748	0.9748	0.9748	0.9748	0.9748	0.9748

## Data Availability

Not applicable.

## References

[1] Boccaletti S., Latora V., Moreno Y., Chavez M., Hwang D.U. (2006). Complex Networks: Structure and Dynamics. Phys. Rep..

[2] Kumar A., Singh S.S., Singh K., Biswas B. (2020). Link Prediction Techniques, Applications, and Performance: A Survey. Physica A.

[3] Gou F., Wu J. (2022). Triad link prediction method based on the evolutionary analysis with IoT in opportunistic social networks. Comput. Commun..

[4] Zhou T. (2021). Progresses and Challenges in Link Prediction. iScience.

[5] Martínez V., Berzal F., Cubero J.C. (2016). A Survey of Link Prediction in Complex Networks. ACM Comput. Surv..

[6] Zhang Q., Tong T., Wu S. (2020). Hybrid Link Prediction via Model Averaging. Physica A.

[7] Mori L., O’Hara K., Pujol T.A., Ventresca M. (2022). Examining Supervised Machine Learning Methods for Integer Link Weight Prediction Using Node Metadata. Entropy.

[8] Newman M.E.J. (2001). Clustering and Preferential Attachment in Growing Networks. Phys. Rev. E.

[9] Jaccard P. (1901). Étude comparative de la distribution florale dans une portion des Alpes et des Jura. Bull. Soc. Vaudoise Sci. Nat..

[10] Barabási A.L., Albert R. (1999). Emergence of Scaling in Random Networks. Science.

[11] Adamic L.A., Adar E. (2003). Friends and Neighbors on the Web. Soc. Netw..

[12] Zhou T., Lü L., Zhang Y.C. (2009). Predicting Missing Links via Local Information. Eur. Phys. J. B.

[13] Salton G., McGill M.J. (1983). Introduction to Modern Information Retrieval.

[14] Aziz F., Gul H., Muhammad I., Uddin I. (2020). Link Prediction Using Node Information on Local Paths. Physica A.

[15] Klein D.J., Randić M. (1993). Resistance Distance. J. Math. Chem..

[16] Brin S., Page L. (1998). The Anatomy of a Large-scale Hypertextual Web Search Engine. Comput. Netw. ISDN Syst..

[17] Jeh G., Widom J. Simrank: A Measure of Structural-context Similarity. Proceedings of the eighth ACM SIGKDD International Conference on Knowledge Discovery and Data Mining.

[18] Lü L., Jin C.H., Zhou T. (2009). Similarity Index based on Local Paths for Link Prediction of Complex Networks. Phys. Rev. E.

[19] Liu W., Lü L. (2010). Link Prediction based on Local Random Walk. Euro. Lett..

[20] Wu Z., Liang Q., Liu Q., Qin Z.G. (2014). Modified Link Prediction Algorithm based on AdaBoost. J. Commun..

[21] Li K., Tu L., Chai L. (2020). Ensemble-model-based Link Prediction of Complex Networks. Comput. Netw..

[22] Ma C., Bao Z.K., Zhang H.F. (2017). Improving Link Prediction in Complex Networks by Adaptively Exploiting Multiple Structural Features of Networks. Phys. Lett. A.

[23] He Y.-L., Liu J.N., Hu Y.-X., Wang X.-Z. (2015). OWA Operator based Link Prediction Ensemble for Social Network. Expert Syst. Appl..

[24] Yu H., Wang S., Ma Q. (2016). Link Prediction Algorithm based on the Choquet Fuzzy Integral. J. Commun..

[25] Li X., Wang Z., Zhang Z. (2022). Complex Embedding with Type Constraints for Link Prediction. Entropy.

[26] Lv H., Zhang B., Hu S., Xu Z. (2022). Deep Link-Prediction Based on the Local Structure of Bipartite Networks. Entropy.

[27] Zhu Y., Liu S., Li Y., Li H. (2022). TLP-CCC: Temporal Link Prediction Based on Collective Community and Centrality Feature Fusion. Entropy.

[28] Leicht E.A., Holme P., Newman M.E.J. (2006). Vertex Similarity in Networks. Phys. Rev. E.

[29] Chebotarev P., Shamis E. (2006). The Matrix-forest Theorem and Measuring Relations in Small Social Groups. arXiv.

[30] Watts D.J., Strogatz S.H. (1998). Collective Dynamics of ‘Small-world’. Netw. Nat..

[31] Vickers M., Chan S. (1981). Representing Classroom Social Structure.

[32] Guimerà R., Danon L., Díaz-Guilera A., Giralt F., Arenas A. (2003). Self-similar community structure in a network of human interactions. Phys. Rev. E.

[33] Von Mering C., Krause R., Snel B., Cornell M., Oliver S.G., Fields S., Bork P. (2002). Comparative assessment of large-scale data sets of protein–protein interactions. Nature.

[34] Pajek Datasets. http://vlado.fmf.uni-lj.si/pub/networks/data/.

[35] Spring N., Mahajan R., Wetherall D., Anderson T. (2004). Measuring ISP Topologies with Rocketfuel. IEEE/ACM Trans. Netw..

[36] Li L., Bai S., Leng M., Wang L., Chen X. (2018). Finding Missing Links in Complex Networks: A multiple-attribute Decision-making Method. Complexity.

[37] Huang X., Zhang L., Wang B., Li F., Zhang Z. (2018). Feature clustering based support vector machine recursive feature elimination for gene selection. Appl. Intell..

[38] Shan N., Li L., Zhang Y., Bai S., Chen X. (2020). Supervised link prediction in multiplex networks. Knowl. Based Syst..

